# Supplementary material for: Exploring the factors behind socioeconomic inequalities in Antenatal Care (ANC) utilization across five South Asian natiaons: A decomposition approach
Source: PLoS One. 2024 Aug 7;19(8):e0304648. doi: 10.1371/journal.pone.0304648 (PMC11305544; doi:10.1371/journal.pone.0304648)
Supplement: S1 Table — (DOCX) [file pone.0304648.s001.docx]

| **S1. Table:** Construct of the independent variables | | |
| --- | --- | --- |
| **Sl. no** | **Variables** | **Groups** |
| 1 | Residential place | Rural, Urban |
| 2 | Maternal Age | 15-24, 25-34, 35-49 |
| 3 | Body Mass Index | <18.50 (Underweight), 18.50-24.90 (Normal), 25.00-29.99 (Overweight), <30 (Obesity) |
| 4 | Women’s Highest Education Level | No education, Primary, Secondary, Higher |
| 5 | Current Working Status | Not working, Working |
| 6 | Husband’s Education Level | No education, Primary, Secondary, Higher |
| 7 | Occupation of the Husband | Agricultural, Non-Agricultural |
| 8 | Wealth Status | Poorest, Poorer, Middle, Richer, Richest |
