# Supplementary material for: Exploring the factors behind socioeconomic inequalities in Antenatal Care (ANC) utilization across five South Asian natiaons: A decomposition approach
Source: PLoS One. 2024 Aug 7;19(8):e0304648. doi: 10.1371/journal.pone.0304648 (PMC11305544; doi:10.1371/journal.pone.0304648)
Supplement: S2 Table — (DOCX) [file pone.0304648.s002.docx]

| **S2. Table:** Factors associated with ANC: Bangladesh | | |
| --- | --- | --- |
| **Characteristics** | | **AOR ANC (95% CI)** |
| **Type of Place of Residence** | |  |
|  | Urban | 1.39 (1.20-1.60)*** |
|  | Rural (RC) |  |
| **Maternal Age** | |  |
|  | 15-24 | 1.15 (0.87-1.53) |
|  | 25-34 | 1.11 (0.84-1.46) |
|  | 35-49 (RC) |  |
| **Body Mass Index** | |  |
|  | <18.50 (Underweight) | 0.73 (0.61-0.87)*** |
|  | 18.50-24.90 (Normal) (RC) |  |
|  | 25.00-29.99 (Overweight) | 1.19 (1.01-1.40)* |
|  | <30 (Obesity) | 1.16 (0.85-1.59) |
| **Women Highest Education Level** | | |
|  | No education (RC) |  |
|  | Primary | 1.79 (1.31-2.45)*** |
|  | Secondary | 2.75 (2.01-3.76)*** |
|  | Higher | 3.54 (2.47-5.07)*** |
| **Respondent Currently Working** | | |
|  | Not working (RC) |  |
|  | Working | 1.37 (1.20-1.56)*** |
| **Husband’s Education Level** | | |
|  | No education (RC) |  |
|  | Primary | 1.11 (0.90-1.36) |
|  | Secondary | 1.39 (1.12-1.73)* |
|  | Higher | 2.15 (1.65-2.82)*** |
| **Occupation of the Husband** | |  |
|  | Agricultural (RC) |  |
|  | Non-Agricultural | 1.12 (0.95-1.32) |
| **Wealth Status** | |  |
|  | Poorest (RC) |  |
|  | Poorer | 1.22 (1.01-1.48)* |
|  | Middle | 1.49 (1.22-1.82)*** |
|  | Richer | 1.56 (1.27-1.92)*** |
|  | Richest | 2.38 (1.87-3.03)*** |

**p<0.05; **p<0.01; ***p<0.001*
