# Supplementary material for: Exploring the factors behind socioeconomic inequalities in Antenatal Care (ANC) utilization across five South Asian natiaons: A decomposition approach
Source: PLoS One. 2024 Aug 7;19(8):e0304648. doi: 10.1371/journal.pone.0304648 (PMC11305544; doi:10.1371/journal.pone.0304648)
Supplement: S3 Table — (DOCX) [file pone.0304648.s003.docx]

| **S3.** **Table:** Factors associated with ANC: India | | | |
| --- | --- | --- | --- |
| **Characteristics** | | **AOR ANC (95% CI)** |  |
| **Type of Place 0f Residence** | |  |  |
|  | Urban | 1.13 (1.06-1.22)** |  |
|  | Rural (RC) |  |  |
| **Maternal Age** | |  |  |
|  | 15-24 | 1.02 (0.93-1.12) |  |
|  | 25-34 | 1.07 (0.98-1.16) |  |
|  | 35-49 (RC) |  |  |
| **Body Mass Index** | |  |  |
|  | <18.50 (Underweight) | 1.03 (0.97-1.10) |  |
|  | 18.50-24.90 (Normal) (RC) |  |  |
|  | 25.00-29.99 (Overweight) | 1.16 (1.07-1.25)*** |  |
|  | <30 (Obesity) | 1.29 (1.13-1.49)*** |  |
| **Women Highest Education Level** | | |  |
|  | No education (RC) |  |  |
|  | Primary | 1.23 (1.12-1.35)*** |  |
|  | Secondary | 1.46 (1.35-1.57)*** |  |
|  | Higher | 1.59 (1.42-1.78)*** |  |
| **Respondent Currently Working** | | |  |
|  | Not working (RC) |  |  |
|  | Working | 1.11 (1.04-1.18)* |  |
| **Husband’s Education Level** | | |  |
|  | No education (RC) |  |  |
|  | Primary | 1.17 (1.06-1.29)* |  |
|  | Secondary | 1.25 (1.15-1.36)*** |  |
|  | Higher | 1.16 (1.03-1.30)* |  |
| **Occupation of the Husband** | |  |  |
|  | Agricultural (RC) |  |  |
|  | Non-Agricultural | 1.04 (0.98-1.12) |  |
| **Wealth Status** | |  |  |
|  | Poorest (RC) |  |  |
|  | Poorer | 1.31 (1.22-1.41)*** |  |
|  | Middle | 1.82 (1.68-1.97)*** |  |
|  | Richer | 2.14 (1.95-2.34)*** |  |
|  | Richest | 2.44 (2.18-2.72)*** |  |

**p<0.05; **p<0.01; ***p<0.001*
