# Supplementary material for: Exploring the factors behind socioeconomic inequalities in Antenatal Care (ANC) utilization across five South Asian natiaons: A decomposition approach
Source: PLoS One. 2024 Aug 7;19(8):e0304648. doi: 10.1371/journal.pone.0304648 (PMC11305544; doi:10.1371/journal.pone.0304648)
Supplement: S4 Table — (DOCX) [file pone.0304648.s004.docx]

| **S4. Table:**  Factors associated with ANC: Nepal | | | |
| --- | --- | --- | --- |
| **Characteristics** | | **AOR ANC (95% CI)** |  |
| **Type of Place 0f Residence** | |  |  |
|  | Urban | 1.23 (0.99-1.53) |  |
|  | Rural (RC) |  |  |
| **Maternal Age** | |  |  |
|  | 15-24 | 1.19 (0.80-1.75) |  |
|  | 25-34 | 0.99 (0.69-1.43) |  |
|  | 35-49 (RC) |  |  |
| **Body Mass Index** | |  |  |
|  | <18.50 (Underweight) | 0.80 (0.61-1.06) |  |
|  | 18.50-24.90 (Normal) (RC) |  |  |
|  | 25.00-29.99 (Overweight) | 1.16 (0.82-1.62) |  |
|  | <30 (Obesity) | 1.49 (0.66-3.37) |  |
| **Women Highest Education Level** | | |  |
|  | No education (RC) |  |  |
|  | Primary | 1.76 (1.32-2.36)*** |  |
|  | Secondary | 2.71 (2.02-3.62)*** |  |
|  | Higher | 8.14(4.81-13.79)*** |  |
| **Respondent Currently Working** | | |  |
|  | Not working (RC) |  |  |
|  | Working | 1.46 (1.17-1.83)** |  |
| **Husband’s Education Level** | | |  |
|  | No education (RC) |  |  |
|  | Primary | 1.23 (0.88-1.72) |  |
|  | Secondary | 1.36 (0.98-1.88) |  |
|  | Higher | 1.20 (0.77-1.88) |  |
| **Occupation of the Husband** | |  |  |
|  | Agricultural (RC) |  |  |
|  | Non-Agricultural | 1.26 (0.97-1.63) |  |
| **Wealth Status** | |  |  |
|  | Poorest (RC) |  |  |
|  | Poorer | 1.20 (0.89-1.62) |  |
|  | Middle | 1.41 (1.04-1.92)* |  |
|  | Richer | 1.78 (1.27-2.50)** |  |
|  | Richest | 2.12 (1.28-3.52)* |  |

**p<0.05; **p<0.01; ***p<0.001*
