# Supplementary material for: Exploring the factors behind socioeconomic inequalities in Antenatal Care (ANC) utilization across five South Asian natiaons: A decomposition approach
Source: PLoS One. 2024 Aug 7;19(8):e0304648. doi: 10.1371/journal.pone.0304648 (PMC11305544; doi:10.1371/journal.pone.0304648)
Supplement: S5 Table — (DOCX) [file pone.0304648.s005.docx]

| **S5.** **Table:** Factors associated with ANC: Pakistan | | |
| --- | --- | --- |
| **Characteristics** | | **AOR ANC (95% CI)** |
| **Type of Place 0f Residence** | |  |
|  | Urban | 1.47 (1.22-1.78)*** |
|  | Rural (RC) |  |
| **Maternal Age** | |  |
|  | 15-24 | 1.10 (0.85-1.42) |
|  | 25-34 | 1.37 (1.10-1.70)* |
|  | 35-49 (RC) |  |
| **Body Mass Index** | |  |
|  | <18.50 (Underweight) | 0.99 (0.72-1.38) |
|  | 18.50-24.90 (Normal) (RC) |  |
|  | 25.00-29.99 (Overweight) | 1.24 (1.01-1.52)* |
|  | <30 (Obesity) | 1.05 (0.82-1.35) |
| **Women Highest Education Level** | | |
|  | No education (RC) |  |
|  | Primary | 1.48 (1.15-1.90)* |
|  | Secondary | 2.33 (1.84-2.96)*** |
|  | Higher | 4.35 (3.07-6.16)*** |
| **Respondent Currently Working** | | |
|  | Not working (RC) |  |
|  | Working | 1.02 (0.77-1.36) |
| **Husband’s Education Level** | | |
|  | No education (RC) |  |
|  | Primary | 1.32 (1.01-1.74)* |
|  | Secondary | 1.44 (1.14-1.82)* |
|  | Higher | 1.43 (1.07-1.91)* |
| **Occupation of the Husband** | |  |
|  | Agricultural (RC) |  |
|  | Non-Agricultural | 0.94 (0.72-1.22) |
| **Wealth Status** | |  |
|  | Poorest (RC) |  |
|  | Poorer | 1.44 (1.10-1.89)* |
|  | Middle | 2.39 (1.78-3.21)*** |
|  | Richer | 2.51 (1.81-3.47)*** |
|  | Richest | 6.76 (4.55-10.03)*** |

****p<0.05; **p<0.01; ***p<0.001***
