# Supplementary material for: Exploring the factors behind socioeconomic inequalities in Antenatal Care (ANC) utilization across five South Asian natiaons: A decomposition approach
Source: PLoS One. 2024 Aug 7;19(8):e0304648. doi: 10.1371/journal.pone.0304648 (PMC11305544; doi:10.1371/journal.pone.0304648)
Supplement: Attachment — Submitted filename: review.docx [file pone.0304648.s006.docx]

**Manuscript Number:** PONE-D-23-19791

**Manuscript title:** Exploring the Factors Behind Socioeconomic Disparities in Antenatal Care (ANC) Utilization across Five South Asian Nations: A Decomposition Approach.

**Journal:** Plos One

**Author:**

This manuscript examines very serious issue of public health as the authors indicate that there is a great variation in the utilization of antenatal care services among the five South Asian Nations. The present work shows that due to variation in the different socio-demographic aspects, the variations in the utilization of antenatal care services also found in the different countries. The manuscript used recent round of nationally representative database to assess the determining factors of utilization of antenatal care services. In my assessment, this manuscript highlighted some important findings which will help for policy implementations. However, the authors are suggested to minor revision in their manuscript. Comments are mentioned below:

1. The authors were taken at least four ANC visits during pregnancy as antenatal care variable in the present study but why not taken full ANC i.e. at least four ANC visits during pregnancy, at least two tetanus injections and at least 100 or more IFA tablets among the different South Asian countries?
2. The authors are suggested to add the rationality for the selection of five South Asian nations in the present study.
3. Women’s autonomy, mass media exposure and male involvement play a vital role for the utilization of antenatal care services among the women but in the present study the authors did not included in their model but why?
4. Discussion section need to be improve in proper manner.
5. I would like to emphasize that the paper needs to check for some typo errors, proofreading, and minor explanations as per the above suggestions.
