# Supplementary material for: Exploring the factors behind socioeconomic inequalities in Antenatal Care (ANC) utilization across five South Asian natiaons: A decomposition approach
Source: PLoS One. 2024 Aug 7;19(8):e0304648. doi: 10.1371/journal.pone.0304648 (PMC11305544; doi:10.1371/journal.pone.0304648)
Supplement: Attachment — Submitted filename: Response List.docx [file pone.0304648.s007.docx]

Title: Exploring the Factors Behind Socioeconomic Disparities in Antenatal Care (ANC) Utilization across Five South Asian Nations: A Decomposition Approach

Thanks to the editor and respective reviewers for taking care of our paper and insights. In this version, we have tried our best to address the comments you made, we believe this version will satisfy you. We are looking forward to hearing a good news.

**Response to reviewer:**

|  | **5. Review Comments to the Author**  **Reviewer #1:** A section mentioning the Socioeconomic status of each of the population group in the five countries would be helpful. It may be included in the methodology section (Profile of the Study area) as it would set the context of the study. | Thank you for your comment. We have added an additional section including those background information of the selected countries. |
| --- | --- | --- |
|  | **Reviewer #2:** This manuscript examines very serious issue of public health as the authors indicate that there is a great variation in the utilization of antenatal care services among the five South Asian Nations. The present work shows that due to variation in the different socio-demographic aspects, the variations in the utilization of antenatal care services also found in the different countries. The manuscript used recent round of nationally representative database to assess the determining factors of utilization of antenatal care services. In my assessment, this manuscript highlighted some important findings which will help for policy implementations. However, the authors are suggested to minor revision in their manuscript. Comments are mentioned below: | Thank you for your comment and acknowledgment. |
|  | 1. The authors were taken at least four ANC visits during pregnancy as antenatal care variable in the present study but why not taken full ANC i.e. at least four ANC visits during pregnancy, at least two tetanus injections and at least 100 or more IFA tablets among the different South Asian countries? | Thanks for your comment, we have categorized the ANC visit mothers who have taken at least four times ANC schooling accordingly by WHO definition. It is unclear to us or we didn’t find any definition that mother’s have to have at least two tetanus injections and at least 100 or more IFA tablets during pregnancy period. In addition, such information is not available in the DHS data set. |
|  | 2. The authors are suggested to add the rationality for the selection of five South Asian nations in the present study. | Thank you for your comment we have added a section mentioning rationale for selecting those five countries in particular. (page 4) |
|  | 3. Women’s autonomy, mass media exposure and male involvement play a vital role for the utilization of antenatal care services among the women but in the present study the authors did not included in their model but why? | Thank you for your observation, however we have considered women’s working status as an indicator of their autonomy and we found it significant for predicting ANC utilization. However, in our analysis, we encountered limitations in the available DHS data, particularly concerning variables like media exposure, which were incomplete or unavailable, particularly in Pakistan and Nepal. Regrettably, these variables had to be omitted from our study. However, future research endeavors could potentially address and incorporate these issues, thereby enriching our understanding of the complex factors influencing the utilization of Antenatal Care (ANC) services across these countries. |
|  | 4. Discussion section need to be improve in proper manner. | We have tried to refine the discussion sections by incorporating robust supporting arguments and ensuring accurate referencing to existing knowledge. |
|  | 5. I would like to emphasize that the paper needs to check for some typo errors, proofreading, and minor explanations as per the above suggestions. | We have thoroughly proofread this manuscript, diligently addressing and rectifying any potential typographical errors. |
